# Supplementary material for: Metabolic gene alterations impact the clinical aggressiveness and drug responses of 32 human cancers
Source: Commun Biol. 2019 Nov 14;2:414. doi: 10.1038/s42003-019-0666-1 (PMC6856368; doi:10.1038/s42003-019-0666-1)
Supplement: Supplementary file 1 — Supplementary Information [file 42003_2019_666_MOESM1_ESM.pdf]

Supplementary Information

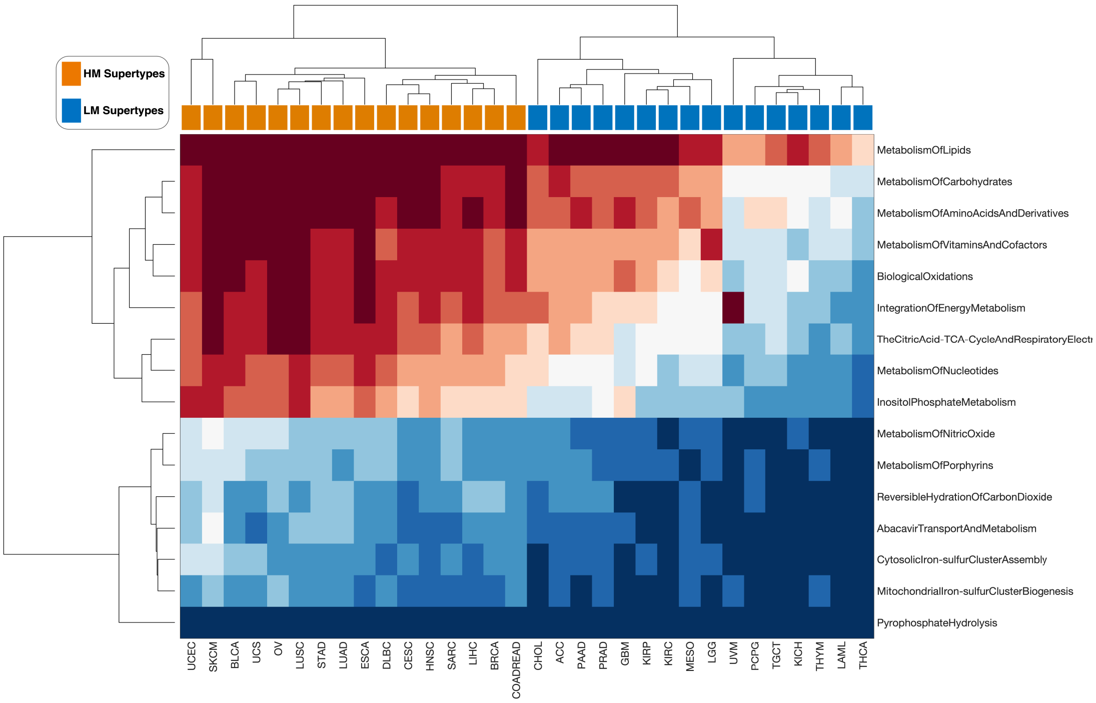

Supplementary Figure 1: Unsupervised hierarchical clustergram of tumours assigned to the two metabolic supertypes of human cancers. The fractions of tumours with altered genes that are involved in each of the 16 first-tier metabolic pathways were used for clustering. The clustergram was produced using the Spearman correlation distance metric with complete linkage.

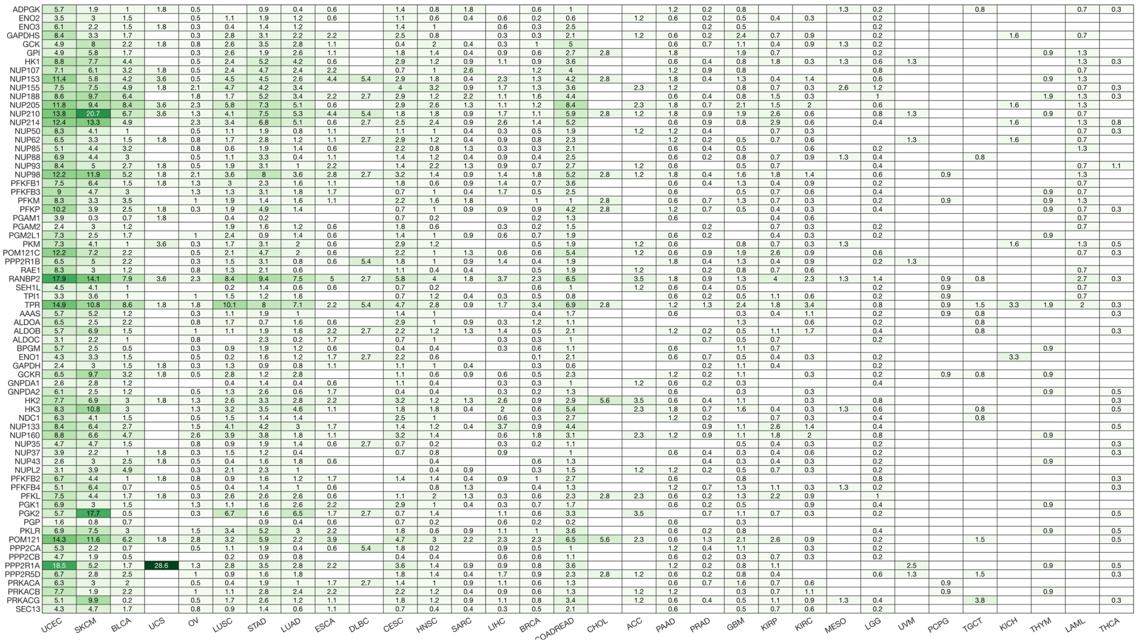

Supplementary Figure 2: Highlight table showing the fractions of tumours with alterations in glycolytic pathway genes across all human cancers. The increasing colour intensities denote higher percentages of altered genes.

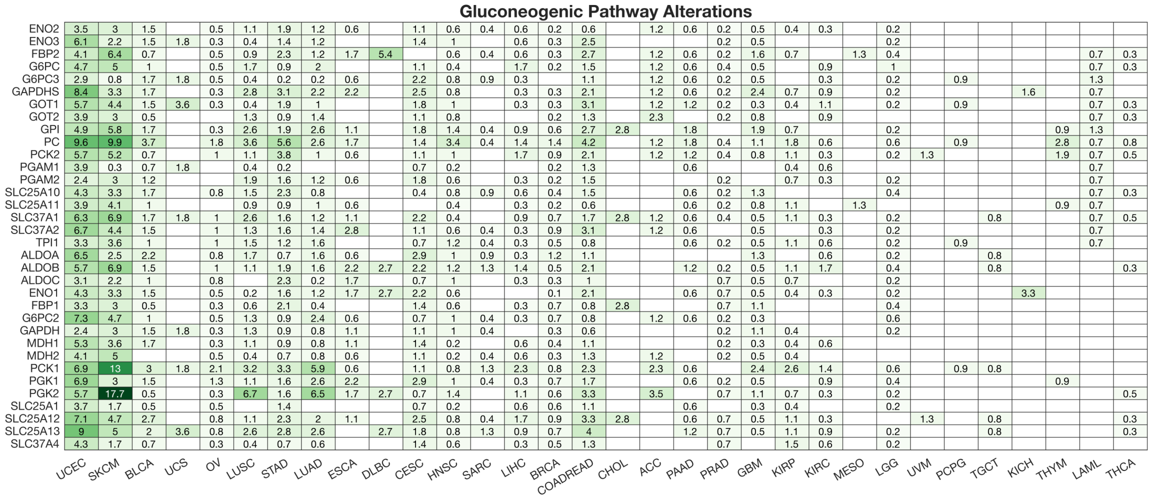

Supplementary Figure 3: Highlight table showing the fractions of tumours with alterations in gluconeogenic pathway genes across all human cancers. Increasing colour intensities denote higher percentages of altered genes.

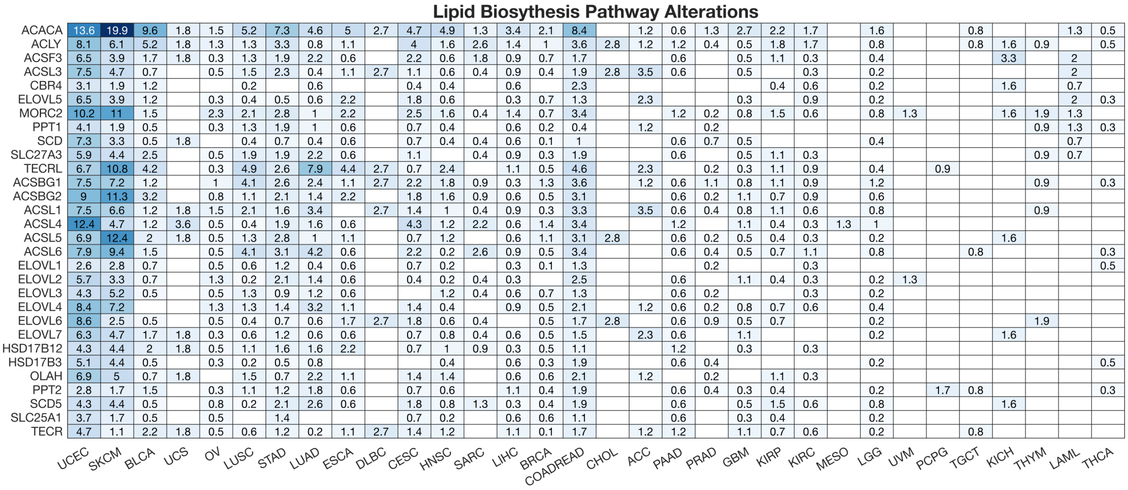

Supplementary Figure 4: Highlight table showing the fractions of tumours with alterations in mitochondrial fatty acid oxidation pathway genes across all human cancers. Increasing colour intensities denote higher percentages of altered genes.

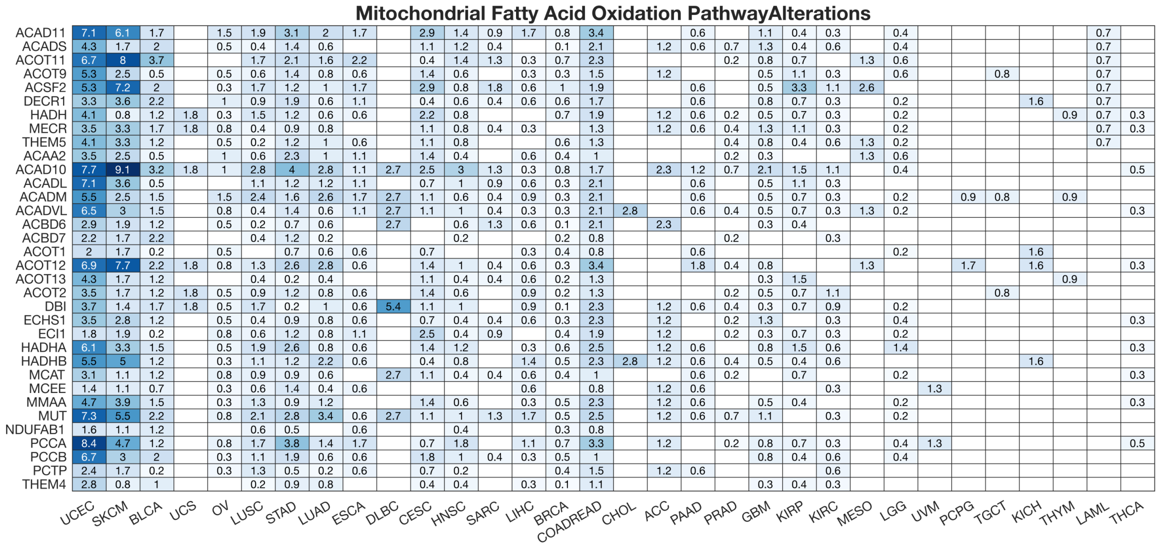

Supplementary Figure 5: Highlight table showing the fractions of tumours with alterations in lipid biosynthesis pathway genes across all human cancers. Increasing colour intensities denote higher percentages of altered genes.

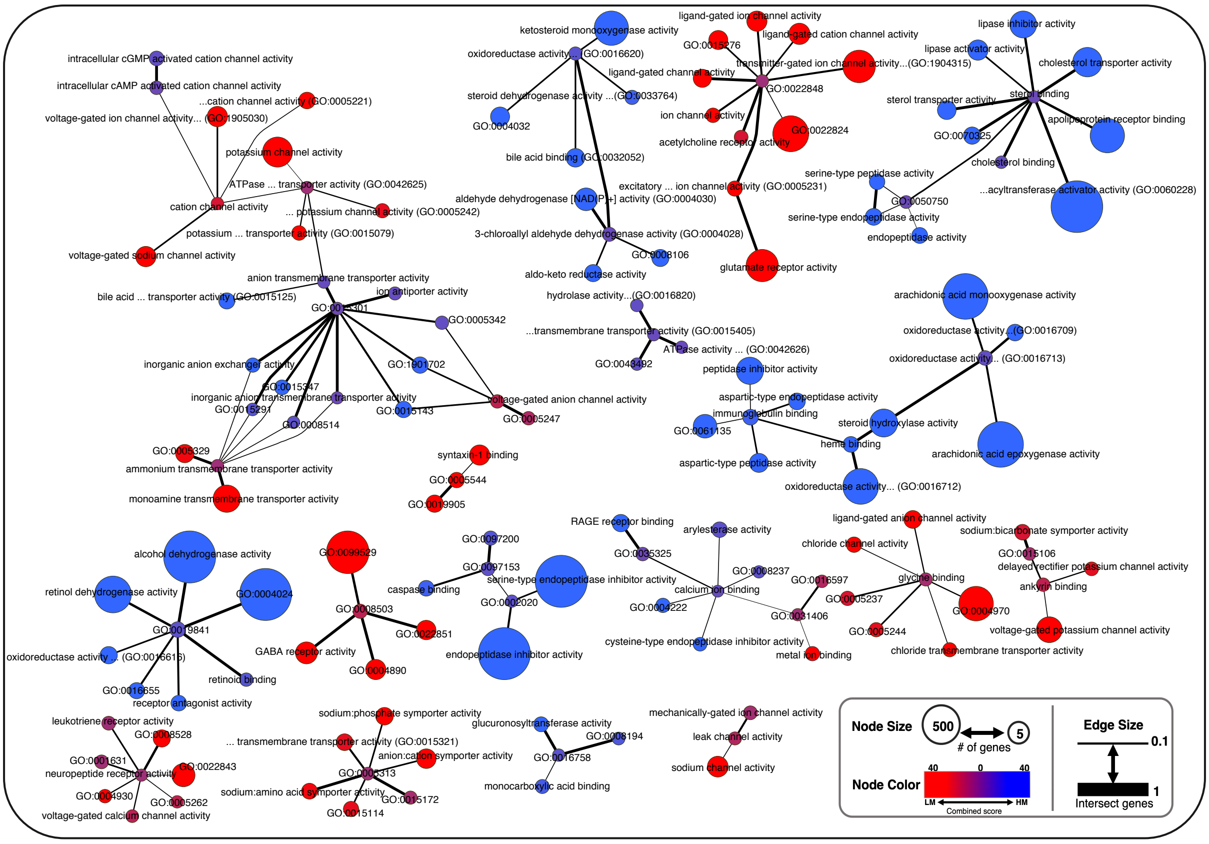

Supplementary Figure 6: Network of Gene Ontology (GO) molecular functions found to be enriched between the HM (high metabolic gene alteration frequencies) and LM (low metabolic gene alteration frequencies) supertypes [1]. Enrichr was used to obtain enriched GO-terms that were visualised in yEd (refer to the methods section; [2]). Each node represents a GO-term with similar nodes clustered together and connected by edges with the number of shared genes between the nodes being represented by the thickness of the edges. The size of each node denotes the gene set size of the represented GO-term. The colour of each node represents the magnitude of the combined enrichment score: red represents enrichment in LM supertype tumours and blue represents enrichment in HM supertypes tumours.

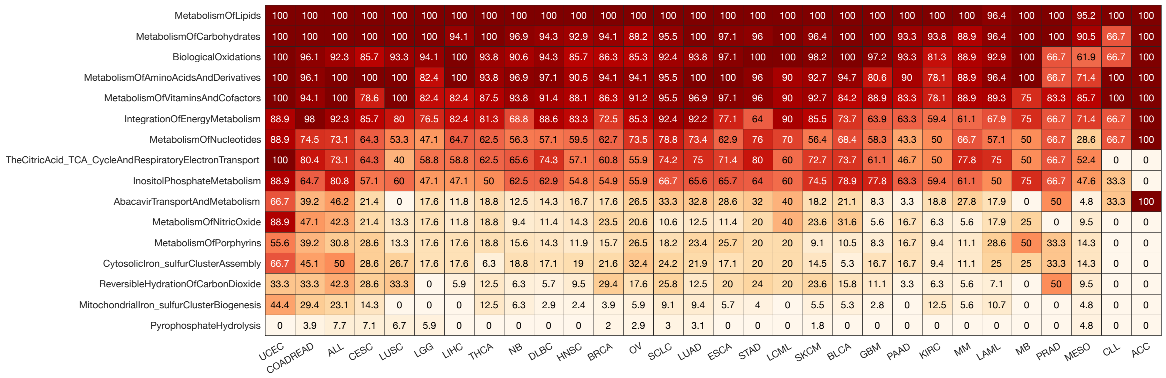

Supplementary Figure 7: Heatmap of Genomics of Drug Sensitivity in Cancer (GDSC) cancer lines showing the percentages of cell lines with alterations to genes involved in each of the 16 first-tier metabolic pathways [3]. Pathways are ordered by decreasing frequencies of gene alterations. Increasing colour intensities denote higher percentages of cell lines with gene alterations.

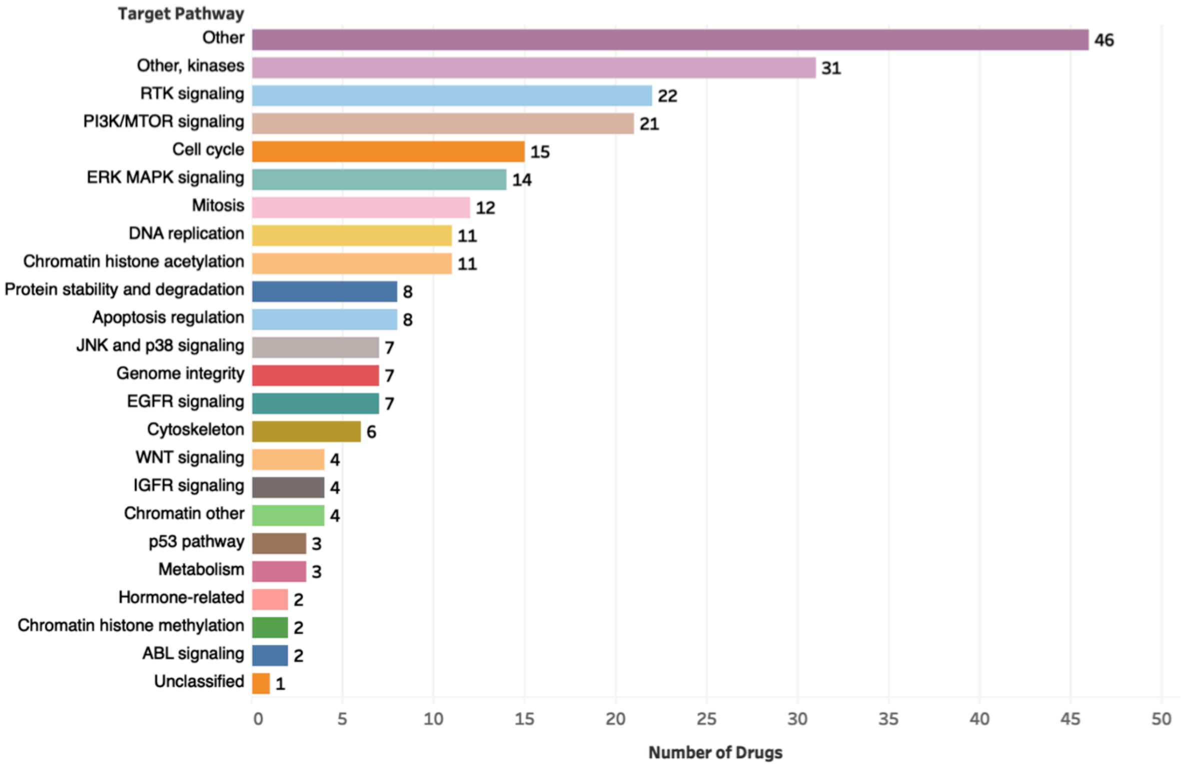

Supplementary Figure 8: The number of anticancer drugs that target 24 signalling pathways and/or biological process that were used by the GDSC to treat cancer cell lines. Colours indicate the targeted pathways.

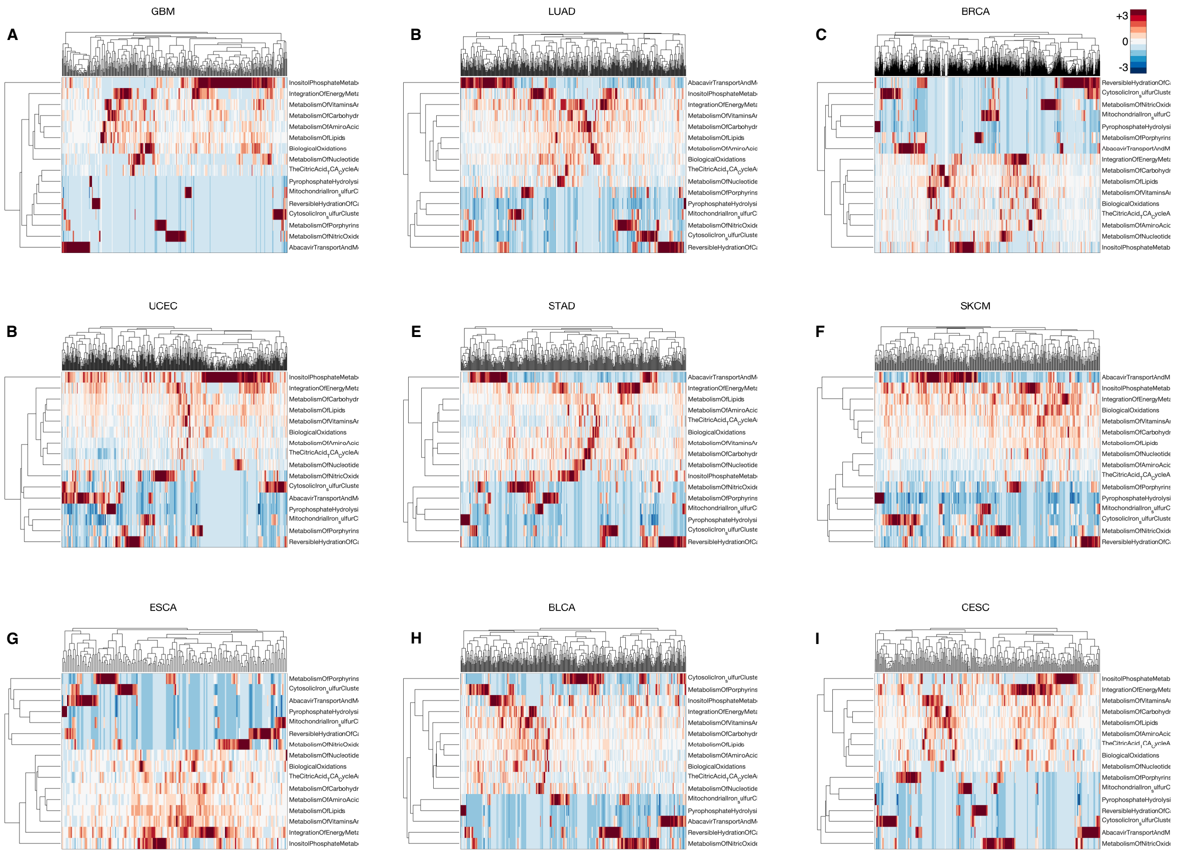

Supplementary Figure 9: Metabolic pathway gene alterations within cancer types. The clustergrams of gene alterations in various human cancer types. Only nine examples of cancer types are shown. The clustergrams show the percentage of tumours with alterations to genes involved in each of the 16 first-tier metabolic pathways.

Supplementary References

1. Gene Ontology Consortium: going forward. *Nucleic Acids Res.* **43**, D1049–D1056 (2015).
2. Chen, E. Y. *et al.* Enrichr: interactive and collaborative HTML5 gene list enrichment analysis tool. *BMC Bioinformatics* **14**, 128 (2013).
3. Yang, W. *et al.* Genomics of Drug Sensitivity in Cancer (GDSC): a resource for therapeutic biomarker discovery in cancer cells. *Nucleic Acids Res.* **41**, D955–D961 (2012).
