# Supplementary material for: Metabolic gene alterations impact the clinical aggressiveness and drug responses of 32 human cancers
Source: Commun Biol. 2019 Nov 14;2:414. doi: 10.1038/s42003-019-0666-1 (PMC6856368; doi:10.1038/s42003-019-0666-1)
Supplement: Supplementary file 2 — Description of Additional Supplementary Files [file 42003_2019_666_MOESM2_ESM.pdf]

Description of Additional Supplementary Files

Supplementary Data 1: Data of cancer studies and genomic alterations: The spreadsheet contains the following results according to the sheet name. TCGA Cancer Types; list and description of The Cancer Genome Atlas (TCGA) studies [1]. GDSC Cancer Types; list and description of Genomics of Drug Sensitivity in Cancer (GDSC) studies [2]. Metabolic Pathways - First Tier; list of and description of first-tier Reactome metabolic pathways. GDSC vs TCGA Alterations; comparison between genomic alterations of GDSC cancer cell lines and TCGA cancers.

Supplementary Data 2: Dose-Response Differences. The spreadsheet contains the following results according to the sheet name. Pathway Drug Response Results; comparison of dose-responses of the HM (high metabolic gene alteration frequencies) and LM (low metabolic gene alteration frequencies) cell lines for all anticancer drugs that target the 24 signalling pathways and/or biological processes. 251 Drug comparisons; comparison of dose-responses of the HM and LM cell lines to each of the 251 anticancer drugs. 41 Sig Drug Results; 41 drugs that showed statistically significant differences in their dose-response comparisons between the HM and LM cancer cell lines. Within Cancer Efficacy Variation; results that show differences in log IC50 values between cancer cell lines of each cancer type with or without alterations to genes involved in each of the 16 first-tier metabolic pathways. Overall Survival Supertypes; within each cancer, comparison of overall survival outcomes between patients with tumours displaying either higher or lower frequencies of alteration to metabolic pathway genes. Disease-Fee Survival

Supertypes; within each cancer, comparison of duration of disease-free survival between patients with either higher or lower metabolic pathway gene alteration frequencies.

Supplementary Data 3: Differential Expression Results; list of statistically significantly differentially expressed genes between the HM and LM cancer supertypes. HM Supertype Upregulated Genes: list of upregulated genes in the HM tumours compared to the LM tumours. LM Supertype Upregulated Genes: list of upregulated genes in the LM tumours compared to the HM tumours. LM Supertype GO Mol Function; enriched gene ontology molecular function in the LM cancer supertypes [3]. HM Supertype GO Mol Function; enriched gene ontology molecular function in the HM cancer supertypes.

References

1. Chang, K. *et al.* The Cancer Genome Atlas Pan-Cancer analysis project. *Nat. Genet.* **45**, 1113–1120 (2013).
2. Yang, W. *et al.* Genomics of Drug Sensitivity in Cancer (GDSC): a resource for therapeutic biomarker discovery in cancer cells. *Nucleic Acids Res.* **41**, D955–D961 (2012).
3. Gene Ontology Consortium: going forward. *Nucleic Acids Res.* **43**, D1049–D1056 (2015).
